# A simple microscopy setup for visualizing cellular responses to DNA damage at particle accelerator facilities

**DOI:** 10.1038/s41598-021-92950-1

**Published:** 2021-07-15

**Authors:** Haibin Qian, Ron A. Hoebe, Michel R. Faas, Marc Jan van Goethem, Emiel R. van der Graaf, Christoph Meyer, Harry Kiewiet, Sytze Brandenburg, Przemek M. Krawczyk

**Affiliations:** 1 Department of Medical Biology, Amsterdam University Medical Centers (Location AMC) and Cancer Center Amsterdam, Amsterdam, The Netherlands; 2 Department of Radiation Oncology, University Medical Center Groningen, University of Groningen, Groningen, The Netherlands

**Keywords:** DNA damage checkpoints, DNA damage response, Double-strand DNA breaks, Fluorescence imaging, Experimental particle physics

## Abstract

Cellular responses to DNA double-strand breaks (DSBs) not only promote genomic integrity in healthy tissues, but also largely determine the efficacy of many DNA-damaging cancer treatments, including X-ray and particle therapies. A growing body of evidence suggests that activation of the mechanisms that detect, signal and repair DSBs may depend on the complexity of the initiating DNA lesions. Studies focusing on this, as well as on many other radiobiological questions, require reliable methods to induce DSBs of varying complexity, and to visualize the ensuing cellular responses. Accelerated particles of different energies and masses are exceptionally well suited for this task, due to the nature of their physical interactions with the intracellular environment, but visualizing cellular responses to particle-induced damage - especially in their early stages - at particle accelerator facilities, remains challenging. Here we describe a straightforward approach for real-time imaging of early response to particle-induced DNA damage. We rely on a transportable setup with an inverted fluorescence confocal microscope, tilted at a small angle relative to the particle beam, such that cells can be irradiated and imaged without any microscope or beamline modifications. Using this setup, we image and analyze the accumulation of fluorescently-tagged MDC1, RNF168 and 53BP1—key factors involved in DSB signalling—at DNA lesions induced by 254 MeV α-particles. Our results provide a demonstration of technical feasibility and reveal asynchronous initiation of accumulation of these proteins at different individual DSBs.

## Introduction

The integrity of genetic material in mammalian cells is constantly threatened by both external and endogenous agents, including UV light, ionizing radiation (IR), mutagenic compounds, (by-products of) metabolic activities and (errors in) DNA processing or replication. All these agents and processes can induce various types of DNA lesions^1,2^. Among them, DNA double-strand breaks (DSBs) are arguably the most severe, frequently leading to cell death, potentially oncogenic mutations or chromosome rearrangements, if not repaired timely and correctly^3^. These dangerous consequences make DSBs a potent, but double-edged sword: on the one hand, DSB-inducing agents power many among the most effective cancer therapies; on the other hand, DSBs can initiate or contribute to the deterioration of genetic material and to carcinogenesis. To counteract these detrimental processes, cells have evolved intricate enzymatic pathways that can efficiently detect, signal and repair DSBs, as well as most other occurring types of DNA lesions.

Emerging evidence indicates that cellular responses to DSBs are at least partly determined by their complexity, i.e. the number, structure and distribution of clustered DNA lesions, such as strand cross-links, base/nucleotide alterations or nicks^4^. For instance, DSBs induced by the clinically-relevant X-rays, characterized by a low linear energy transfer (LET), are generally considered to be relatively simple. On the other end of the complexity spectrum are the DSBs generated by accelerated, high-LET, heavy particles, which are often accompanied by multiple other lesions, including DSBs, often in close (nanometer-scale) proximity^5^. These complex DSBs pose a considerable challenge to the repair machinery, require extensive processing by various enzymes, and have enhanced relative biological effectiveness (RBE) in inducing genomic rearrangements or cell death, as compared to their X-ray-induced counterparts^5–7^. According to a more recent hypothesis, the increased severity of biological effects of high-LET radiation may be a consequence of micrometer-scale DSB clustering^8^.

The differences in cellular responses to simple and complex DSBs are particularly relevant, and somewhat controversial, in the context of therapeutic accelerated protons, which induce simple, as well as more complex DNA lesions^9^. Results of recent studies suggest, for instance, that repair of proton-induced DSBs may require a different enzymatic machinery, as compared to X-ray induced lesions^10–12^. Such insights could be potentially exploited in new therapeutic strategies, combining particle irradiation with small-molecule inhibitors targeting the relevant DSB repair pathways, or aid in selection of patients harboring tumors with increased sensitivity to accelerated particles. However, in spite of these important developments, our understanding of cellular responses to DNA lesions of varying complexity is still limited and methods for studying this aspect of DNA damage response in detail are urgently needed.

One helpful characteristic, shared by most of the proteins known to be involved in DSB repair, is their high affinity for the broken DNA ends and/or for the surrounding chromatin, resulting in the formation of microscopically discernible focal accumulations around DSB sites. First discovered over two decades ago^13,14^, these so-called ‘foci’ are indicative of the proper functioning of repair machinery and have, therefore, become an important biological readout of DSB repair activities that can be visualized and studied using various microscopy techniques^15^. Notably, real-time imaging of cells that express modified, fluorescent fusion proteins has revealed important insights into the functioning of DNA repair in response to various lesions, primarily those induced by visible and UV light^16,17^. Such imaging techniques have also been applied to study the accumulation of repair proteins at sites of DSBs induced by a wide range of IR types and energies^18–40^. Most of these studies are performed at complex particle accelerator facilities and rely on sophisticated microscopy setups, integrated with the beamline hardware. Some advanced implementations allow precise irradiation of cells with a predefined number of particles and/or targeting (sub)cellular structures (reviewed in^41–44^).

Here we describe a simple, inexpensive, transportable, live-cell irradiation and real-time fluorescence imaging confocal microscopy setup that should be compatible with most particle accelerator beamlines. The setup can be paired with any inverted fluorescence microscope, assembled and disassembled within ~ 30 min and allows short- and long-term imaging. In the current proof-of-concept study, we use this setup to analyze the accumulation of signaling proteins MDC1, RNF168 and 53BP1 at DSBs induced by 254 MeV α-particles, in non-cancerous human retinal pigmented epithelium cells. Our results demonstrate the feasibility of the experimental approach and confirm the recently-described asynchronicity of protein accumulation at IR-induced DSBs.

## Results

### The imaging setup

**Figure 1:**
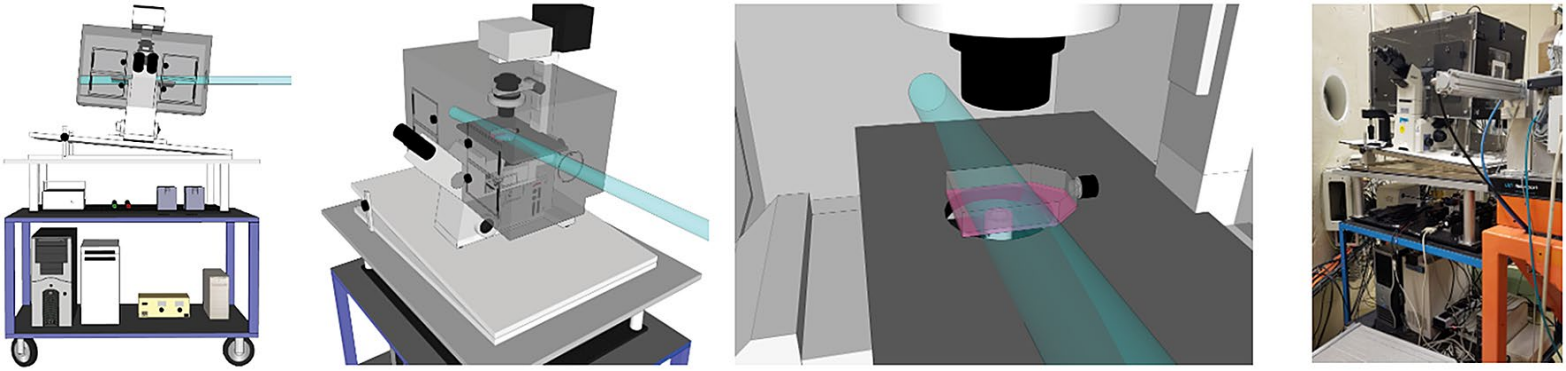
Overview of the imaging setup. Schematic overview of the live-cell microscopy setup at KVI-CART facility in Groningen, The Netherlands. The microscope is positioned on a transportable cart, on a platform at a low angle (~ 5°). The last photograph is the microscopy setup after installation in the beamline.

There are at least two major challenges when performing (real-time) microscopy in a typical particle-accelerator facility. First, the setup of the accelerator beamline must remain flexible to accommodate the different types and arrangements of beam instrumentation, which can change depending on other ongoing experiments. Second, the beam time tends to be very expensive and any beamline setup adjustments required for microscopy should be minimal and quick. To address both challenges, we designed a simple microscopy setup around two concepts. First, all required hardware was fully contained on a small shelved, 4-wheel cart. Second, the inverted confocal microscope and 37 °C incubator were mounted on an adjustable top platform, at a small (~ 5.5°) angle (relative to the horizontal beam, Fig. 1). An interactive version of this and all subsequent figures, as well as all underlying data, are available via (https://create.FiglinQ.com/dashboard/h.qian:15/#/). This simple setup was constructed for under 1000 €, without requiring any major modifications of the microscope or beamline hardware. It can be installed and removed from the beam vault by two operators within ~ 30 min.

### Irradiation and dosimetry

#### Shaping of the radiation field

The irradiations were performed at the in-air station^45^ of the accelerator facility of KVI-CART, University of Groningen using a 360 MeV ^4^He beam. After exiting from the beam line vacuum into air through a 50 µm Aramica foil, the beam was focused to a circular spot with a full width half maximum (FWHM) of about 4 mm at the location of a plane Pb scatter foil with a thickness of 1.16 mm. The angular distribution of the particles after passing through the scatter foil has, according to SRIM simulations^46^, a gaussian shape with a FWHM of about 42 mrad, and is essentially determined by the multiple Coulomb scattering in the foil.

The beam then propagates through air to a field shaping collimator of brass with a thickness of 45 mm (sufficiently thick to stop particles that do not pass through the aperture) located 3 m downstream from the scatter foil, before reaching the irradiation position located 3.65 m downstream from the scatter foil. At the location of the field shaping collimator, the beam has a circular, gaussian shape with a FWHM of about 125 mm. The homogeneity of the dose distribution at the irradiation position was verified with a LANEX™ scintillating screen^47^ and a CCD camera (0.17 mm/pixel at the screen position)^48^. The homogeneity in the central 10 mm of the field corresponding to the cell culture was found to be better than 1%.

#### Dosimetry

The delivered dose is controlled by a large parallel plane beam ionization monitor (BIM) that encompasses the full beam. It is calibrated in terms of dose at the irradiation location along the lines described in the IAEA Internal Code of Practice TRS-398^49^ using a PTW 23,343 Markus ionization chamber with 20 mm polystyrene build-up material in front of it. The calibration of the Markus chamber is traceable to national standards of the German National Metrology Laboratory, PTB Braunschweig.

The dose delivered to the cell culture cannot be determined directly from the calibration measurements because of the differences between the geometry of the setup during the calibration measurements and during the actual irradiation. Therefore, Monte Carlo simulations have been performed with the simulation codes MCNPX^50^ and GATE-GEANT4^51,52^. These simulations include the full irradiation setup, starting at the beamline exit foil and ending at the irradiation position.

The simulations have been performed for two geometries: one used for the actual cell irradiation, and the other one for the dosimetric calibration. The ratio of the delivered dose per starting particle for both geometries yielded a correction factor for the dose calibration obtained from the measurements described above. The correction factors from both simulation codes agree within the statistical uncertainty of the simulations. Systematic uncertainties are assumed to be small because inaccuracies in the models used in the simulation cancel each other out to a large extent: the materials that differ between the two geometries have a comparable atomic number (C_8_H_8_ versus H_2_O) and surface density (approximately 1 g/cm^3^) and thickness (approximately 20 mm).

**Figure 2:**
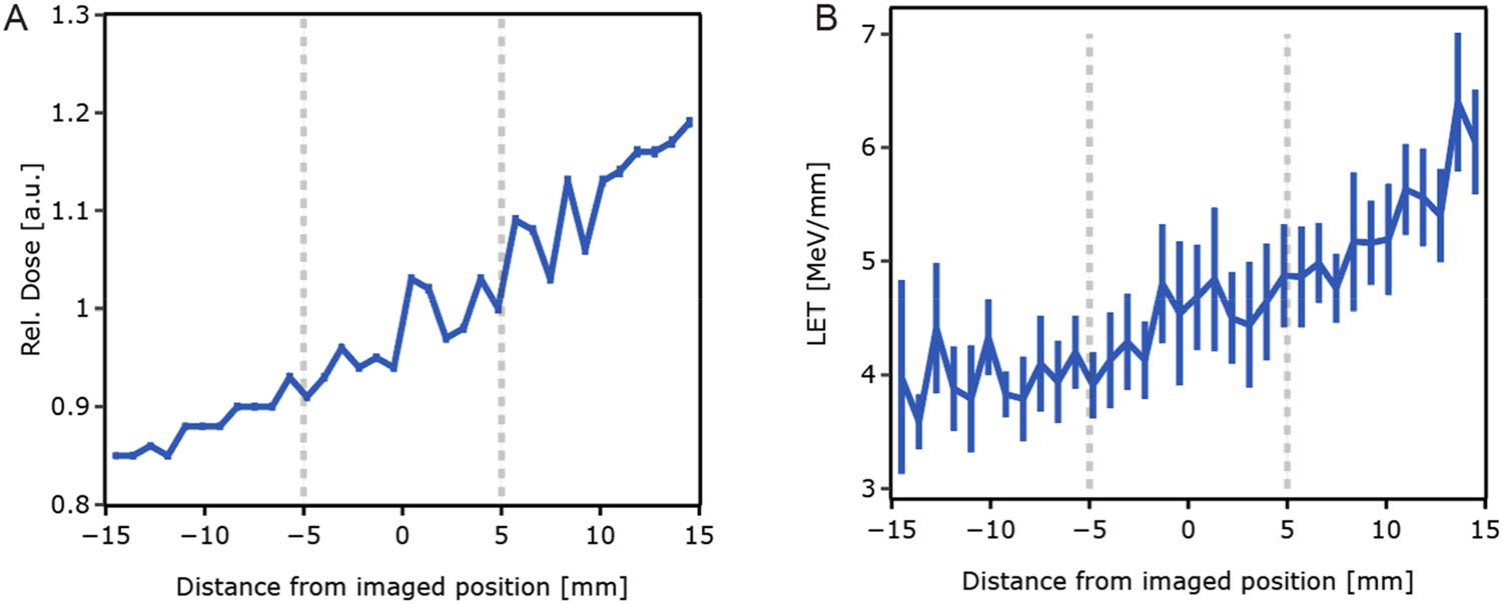
(**A**) Simulated dose distribution across the bottom of the cell culture vessel, in the beam direction, relative to the dose measured with the Markus chamber. (**B**) Simulated dose-averaged LET along the bottom of the cell culture vessel in the beam direction. For both, the origin corresponds to the centre of the imaged glass window. The vertical lines mark the boundary of the window.

According to the Monte Carlo simulations, the dose calibration at the centre of the cell imaging vessel had to be corrected by a factor of 0.986 (± 0.005), as compared to the calibration measurements. The dose delivered to the proximal side of the vessel was approximately 4% lower than the dose delivered to the centre, while the dose delivered to the distal part was ~ 5% higher (Fig. 2A) (https://create.FiglinQ.com/dashboard/h.qian:15/#/). The α-particles at the centre of the vessel had an energy of 254 MeV. The dose-averaged LET was found to be 4.54 MeV/mm. LET increased from 4.4 to 4.9 MeV/mm over the length of the images part of the vessel (Fig. 2B).

### Proof-of-concept experiments: visualizing the accumulation of MDC1, RNF168 and 53BP1 at α-particle induced DSBs

To validate our experimental approach, we have performed real-time imaging of accumulation of fluorescently tagged MDC1, RNF168 and 53BP1 in human retinal pigmented epithelium cells (ARPE-19). During the cellular response to DSB induction, the lesions are likely first detected by the KU70/80 heterodimer and by the MRE11-RAD50-NBS1 complex, with the latter serving as an activator of the ATM kinase. Activated ATM then phosphorylates the histone variant H2AX, resulting in the so-called γH2AX, in nucleosomes within 1–2 million base pairs around the DSB site^13^. γH2AX is bound by MDC1, which forms a platform for subsequent recruitment of additional ATM molecules, and the E3 ubiquitin ligases RNF8 and RNF168. The ubiquitin chains, deposited by RNF8/RNF168 on the neighbouring histones, attract downstream proteins, such as 53BP1 or BRCA1, which then activate and control proteins that physically repair the broken DNA, generally via different variants of either the homologous recombination or the non-homologous end joining pathway^53^.

**Figure 3:**
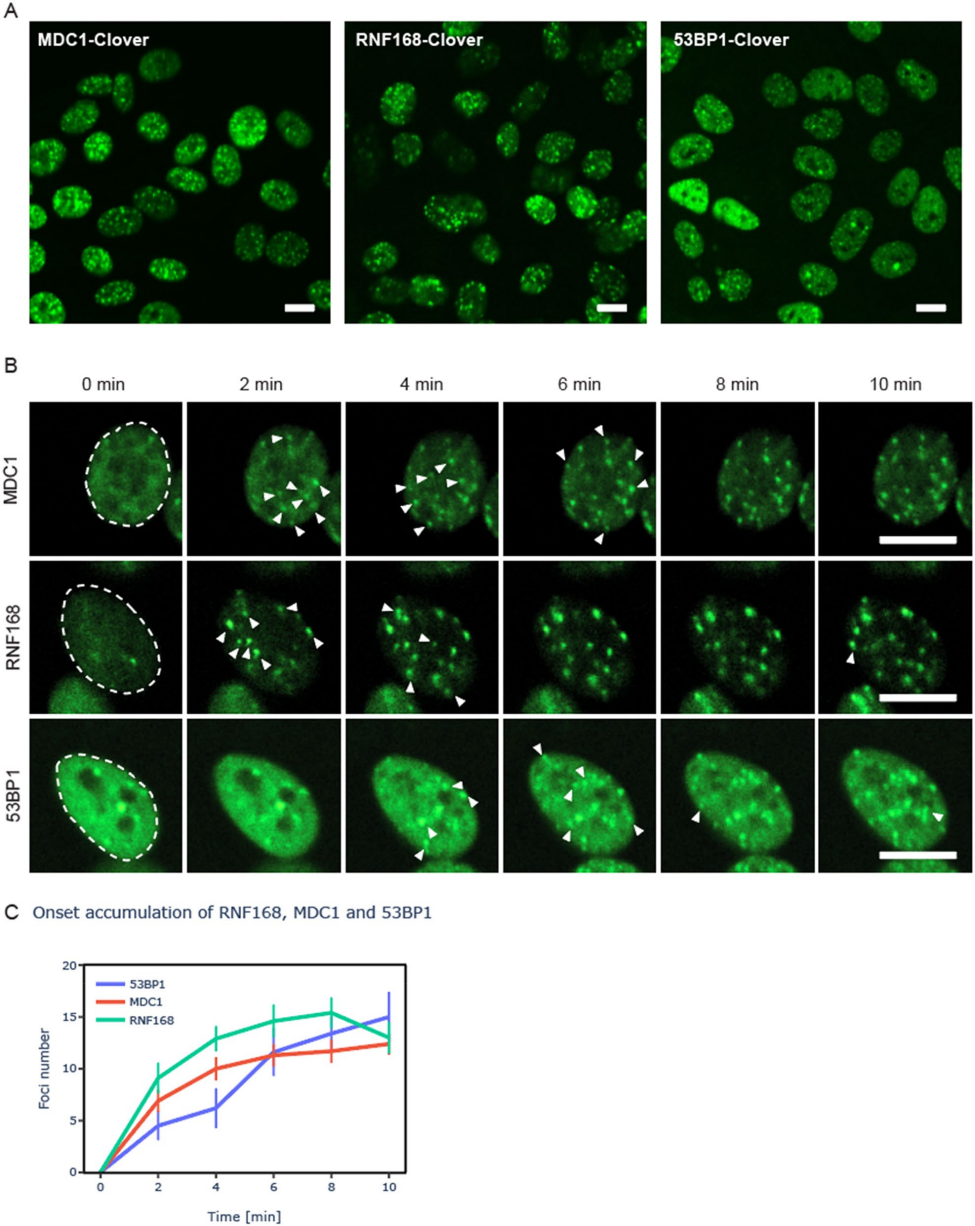
Asynchronous accumulation of MDC1, RNF168 and 53BP1 at α-particle induced DSBs. ARPE19 cells expressing the indicated fluorescently-tagged proteins have been irradiated with ~ 1 Gy of 360 MeV α-particles and imaged for 16 min at 2-min intervals. (**A**) Overview of a single imaged field 10 min after irradiation. All images were 3D scans, processed by deconvolution and then converted into a single maximum intensity projection. (**B**) Galleries showing projections of individual 3D images acquired at the indicated time-points. The white arrows indicate newly appearing foci. (**C**) A number of foci per cell as a function of time. At least 30 cells have been analyzed per data point. Error bars: standard error of the mean (SEM). Scale bar: 10 μm.

MDC1, RNF168 and 53BP1 thus all participate in the early stages of DSB responses, forming clear, microscopically-discernible foci at chromatin surrounding each DSB within minutes after damage induction (Fig. 3A) (https://create.FiglinQ.com/dashboard/h.qian:15/#/), and are thus particularly amenable for real-time imaging^27^. To observe their accumulation at DSB sites in real-time, we exposed cells expressing fluorescently-tagged versions of these proteins to 1.8 ± 0.1 Gy of 254 MeV α-particles (irradiation duration ~ 2 s) and then acquired images for the next 16 min, at 2-min intervals. All proteins showed the expected accumulation pattern (Fig. 3B), with first foci appearing within 2 min after irradiation. We then noted, for each cell, the time of the first appearance of each detected focus, and plotted the fraction of all foci detected at each time point (Fig. 3C). Interestingly, our results show that the onset of foci accumulation is not synchronous, with new foci continuing to appear for up to ~ 10 min after irradiation.

**Figure 4:**
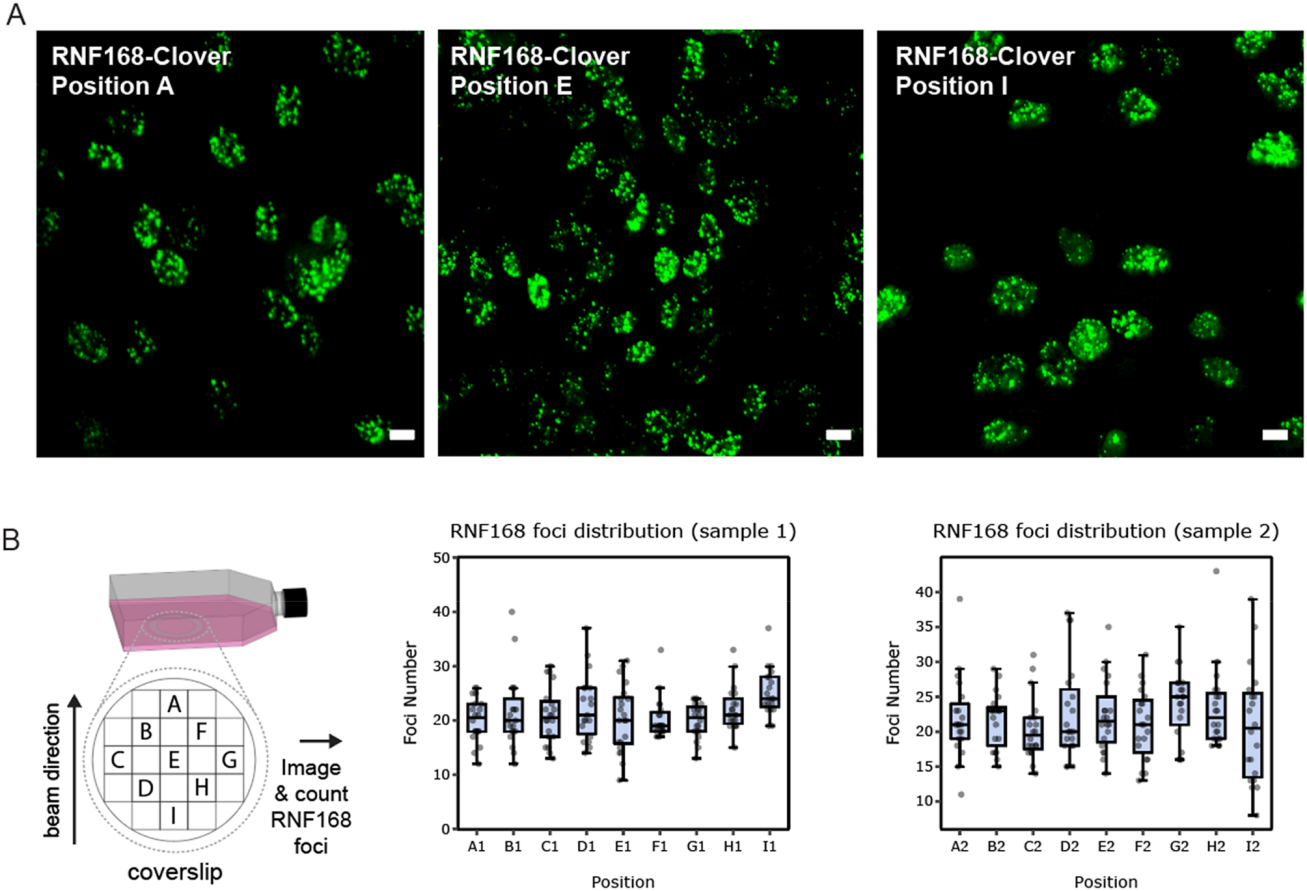
RNF168-Clover foci distribution across the imaged areas of the cell culture vessels. After acquiring time-lapse movies, cells described in the legend in Fig. 3 were fixed and imaged in the indicated areas of two different vessels. Next, the number of RNF168 foci per cell was determined semi-automatically. All images were 3D scans, processed by deconvolution and then converted into a single maximum intensity projection. (**A**) Representative image of an irradiated area. Scale bar: 10 μm. (**B**) Quantification of the average number of RNF168 foci in each area confirms uniform dose distribution. The beam direction is marked with an arrow. 20 cells have been analyzed per area. Error bars: data range.

To determine whether the entire surface of the imaged vessel area was irradiated uniformly, we fixed the irradiated cells expressing RNF168-Clover and acquired 3D images of cells in different areas of two different irradiated vessels (Fig. 4A) (https://create.FiglinQ.com/dashboard/h.qian:15/#/). We then manually counted the number of RNF168 foci in each cell and found that these numbers were comparable in all analysed areas (Fig. 4B), confirming uniform dose distribution. On average, we detected ~ 21 foci per cell.

## Discussion

**Figure 5:**
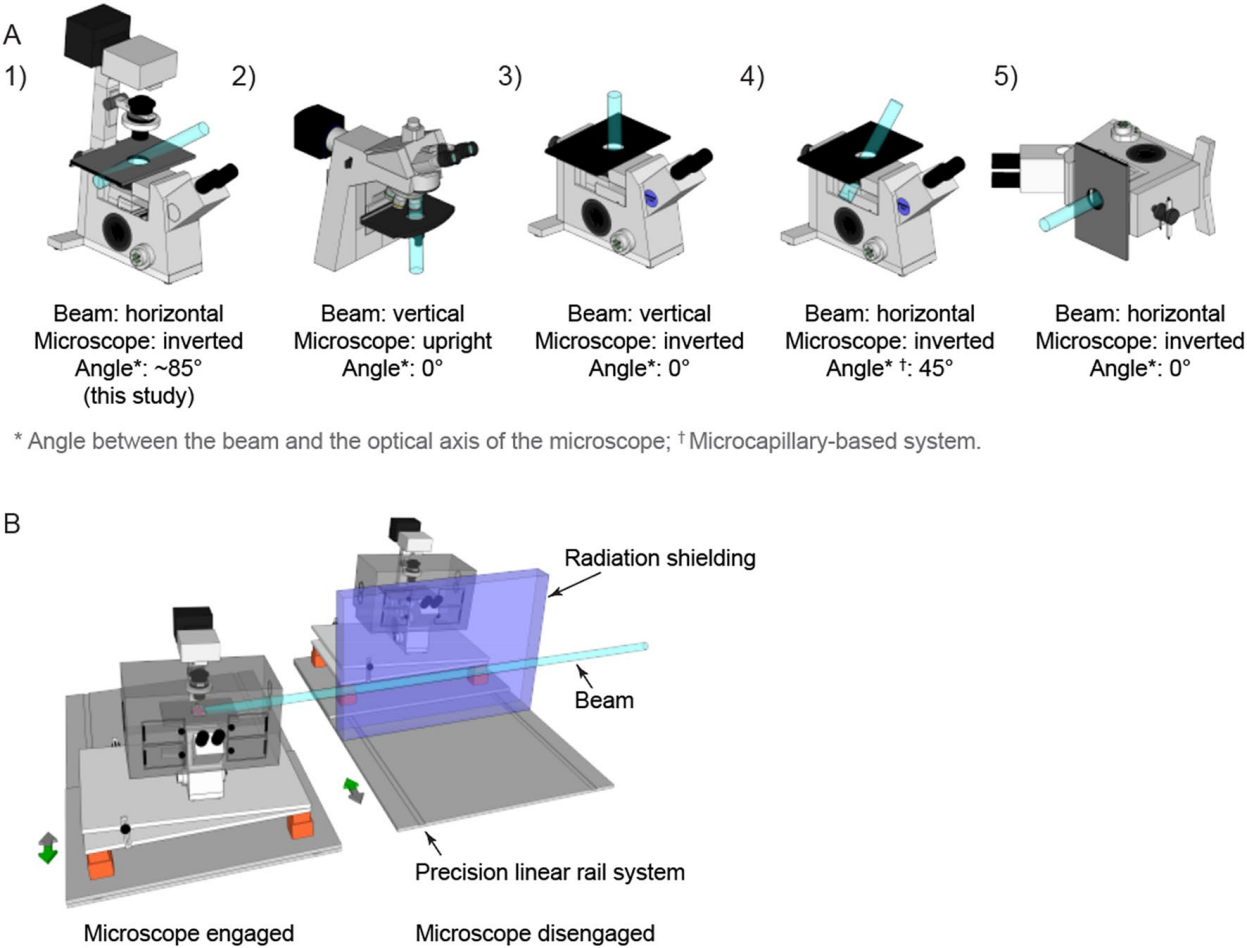
Different arrangements of the microscope, relative to the particle beam, in existing imaging setups at particle accelerator facilities. (**A**) Schematic illustration of the existing arrangements, with references to the relevant publications. (1) this study; (2) refs^18,31,32,35,38^; (3) Refs.^22,34,40^; (4) Ref.^37^; (5) Refs.^9,19,23,36,39,54,55^. (**B**) Considered future automated microscopy setup at the KVI-CART facility in Groningen, The Netherlands.

In this short report, we describe a simple and inexpensive microscopy setup for real-time fluorescence imaging of cellular responses to particle-induced DSBs. Our approach is based on a nearly perpendicular arrangement of the microscope optical axis relative to the beamline, contrasting with most described microscopy setups (Fig. 5A) (https://create.FiglinQ.com/dashboard/h.qian:15/#/), in which the optical axis is generally aligned with the beam path. This difference leads to some drawbacks, but also important advantages.

Perhaps the biggest disadvantage of our approach is the difficulty in determining and controlling the length of the path that the accelerated particles travel in the cell culture vessel and its (plastic) walls, before reaching the targeted cells (Fig. 1A). In beam-aligned microscope arrangements (with 0° angle between the beam and the optical axis of the microscope), where particles enter the culture vessel from the top or bottom, this length can be minimized by using vessels with a low volume of medium and thin lids/bottoms, which allows precise dose calculation and delivery, as well as targeted irradiation of subcellular compartments, in some advanced facilities paired with the ability to irradiate individual cells with predefined particle numbers^41–44^. Our Monte Carlo simulations of the beam transport inside the culture dishes indeed show that the particle count and energy can be considerably different in different locations of the imaged vessel area, especially for low-energy particles that will be stopped by the cell culture vessel wall and medium therein. The slightly angled walls of the used vessels are an additional complication in simulations, as well as in performing precise dosimetry.

Among the advantages of our approach are flexibility, low cost and simplicity. The beam-perpendicular arrangement (with particle beam at near-90° angle to the optical axis of the microscope) relies on a standard, unmodified microscope and on a transportable platform that, with a footprint of ~ 1 m^2^, should be compatible with most existing accelerator facilities. Beam-aligned arrangements, in contrast, require either beam bending, which is costly, complex, and may be incompatible with accelerator facility layouts, or—more often—rotating the microscope by 90°. The latter approach, adopted by multiple facilities (Fig. 5A5), requires a robust, heavy-weight support structure, as well as major, destructive modifications of the microscope body, since all beam-blocking parts must be permanently removed. These modifications often render the microscope incapable of transmitted light imaging, which is essential for non-fluorescent samples. Moreover, such perpendicular arrangements or modifications may be not recommendable for some microscopes and require customized, water-tight cell imaging chambers, since gravity would otherwise cause the draining of the culture medium. Perpendicular setups may also be more time-consuming to assemble in the beamline, although one can also mount them permanently on a transportable cart, similar to our approach, to reduce installation time.

The presented set of proof-of-concept experiments was limited by using an older-generation microscope model that is characterized by relatively slow image acquisition and lack of auto-focusing hardware, such that the focus needed to be maintained manually by microscope operators. Even with this setup, however, we were able to image the accumulation of three different DSB signalling proteins after 254 MeV α-particle irradiation. Our results confirm the recently reported non-synchronous foci formation that was previously detected when observing the accumulation of 53BP1 proteins around DSBs induced by X-rays and accelerated heavy ions^54^. Nevertheless, the interpretation of these results is difficult because of our inability to image the entire 3D volume of cell nuclei, and also because of the relatively low time resolution (2 min) that was chosen in our proof-of-concept experiments. We are currently investigating the mechanisms driving this highly interesting phenomenon using our ultra-soft X-ray irradiator^27^.

In the future, we plan to upgrade our imaging setup based on a state-of-the-art microscope that will allow fast, multi-channel imaging of two- and three-dimensional cell cultures (e.g. organoids or spheroids). Further, we will investigate the feasibility of a motorized platform on a permanent rail system that would allow fast and precise relocation of the microscope from a radiation-shielded area of the bunker into a predetermined position in the beamline. This would allow prolonged imaging sessions and optimized use of spare beam-time during other experiments, without the need of (dis)assembly (Fig. 5B).

## Methods

### Microscopy setup

The microscope was fixed on an angled platform, resting, via rubber dampers, on an optical breadboard, which itself also rested on additional dampers to provide maximum resistance to vibrations. The height of the platform was chosen such that the tip of the objective in its working position was located in the centre of the beam cross-section. The microscope was aligned relative to the beam using three laser planes materializing the XY, XZ and YZ planes, where the Z-axis corresponds to the beam axis, and an additional laser along the Z-axis. The positioning accuracy of the microscope was better than 1 mm in all three directions. This accuracy is more than sufficient given the 20 mm width of the radiation field and the 10 mm width of the imaged coverslip. To allow the penetration of the beam into the microscope imaging area, a circular opening was cut out in the perspex incubator wall perpendicular to the beam and sealed with a 12.5 μm-thick mylar foil (Fig. 1A). Once samples were positioned on the microscope stage, the microscope was operated remotely from the accelerator control room, via a wired networking connection, using standard Windows Remote Desktop software, such that imaging could be started in sync with irradiation and the microscope controlled in real-time. Cells were cultured and imaged in modified 25 cm^2^ vessels (T25 cell culture flasks, Fig. 4B) on glued-in glass coverslips for optimal optical performance.

### Irradiation and dosimetric calibration

The irradiation was performed with a 360 MeV α-particle beam delivered by the AGOR cyclotron at the KVI-Center for Advanced Radiation Technology (KVI-CART) of the University of Groningen. The irradiation beamline used for the experiment is described in more detail in ref^45^. The beam was focused to a spot with a full-width half maximum of about 4 mm at the location of a 1.16 mm thick lead scatter foil. The radiation field at the irradiation position 3.65 m downstream from the scatter foil is shaped with a 45 mm thick brass collimator positioned 3.00 m downstream from the scatter foil. For the dose calibration, a 70 mm circular collimator was used, while a 20 × 20 mm^2^ square collimator was used for the actual irradiation. The radiation field in the central 10 mm corresponding to the width of the imaged cell culture dish surface, imaged with a LANEX™ scintillation screen (LANEX Screens) and a CCD chamber, is homogeneous to better than 1% (Figure S1) (https://create.FiglinQ.com/dashboard/h.qian:15/#/).

The dose administered during the irradiation was controlled with a parallel plate beam ionisation monitor (BIM) of 100 mm diameter, mounted 1.60 m downstream from the scatter foil. The beam passing through this ionisation chamber is collimated to a diameter of 66 mm by upstream collimators. The BIM is calibrated according to the procedure described in the IAEA International Code of Practice TRS-398^49^ by measuring the absolute dose at the irradiation position with a Markus Chamber (type 23,343, PTW, Freiburg) in a 70 mm diameter field to ensure that the Bragg-Gray condition is met. To approximate the water layer at the imaged location of the cell culture dish, a 20 mm thick polystyrene plate was placed in front of the Markus chamber. The systematic error of this calibration for heavy ion beams is in TRS-398^49^ estimated to be 3.4%.

### Monte Carlo simulations

The geometry of the cell culture vessel is such that direct, precise measurement of the dose delivered to the imaged location is not feasible. Therefore, Monte Carlo simulations with MCNPX^50^ and GATE-GEANT4^51,52^ were performed to establish the relation between the dose measured with a calibrated PTW 23343 Markus ionization chamber at the irradiation location, and the dose delivered to the cell culture. In both simulations, the configuration of the complete beamline as used for the calibration and the actual irradiations, respectively, was implemented. The detailed geometries for the Markus chamber and for the imaging cell culture vessel are displayed in Figure S2 (https://create.FiglinQ.com/dashboard/h.qian:15/#/). The Markus chamber was modelled as a 30 mm diameter cylinder filled with water. The deposition dose was scored in its sensitive volume of 5.3 mm in diameter and 2 mm thickness, located 1.06 mm (water equivalent thickness of the window) from its front face.

The following particles were tracked in the simulations:^4^He; ^3^He; ^1^H; ^3^H; neutrons; electrons and photons. Particles were tracked as long as their energy exceeded the following limits: 0 eV for neutrons; 1 keV for electrons and photons; 1 MeV/amu for the hydrogen and helium isotopes. In the MCNPX simulations standard settings of the program were used, with the exception of the EFAC setting, which was increased from its default value of 0.917 to 0.99 in order_BERT_HP physics list was used.

The simulation of the radiation transport through the beamline was started at the 50 µm Aramica exit foil, through which the beam passes from the beamline vacuum into the air. The beam of 360 MeV α-particles was defined to have a gaussian shape with an FWHM of 4 mm, of which the tails were cut in the MCNPX simulation, and a zero divergence. This definition of the initial phase space of the beam has essentially no impact on the simulated field shape at the irradiation position. The field shape is almost entirely (> 99%) determined by the multiple scattering in the 1.16 mm Pb scatter foil, mounted 100 mm downstream from the exit foil. After passing through the scatter foil, the beam propagates through 3 m of air to the field-shaping collimators (45 mm thick brass). Along this path, several brass collimators with sufficient thickness to stop particles incident on their front face have been installed to intercept particles that would anyway not contribute to the radiation field. The beam also passes through the ionization monitor used to control the irradiation. In the simulation, this ionization monitor is modelled as a single aluminium foil of 55 µm thickness.

In the simulations, multiple scattering in the air, in the various collimators, and the nuclear interaction in all materials traversed, was taken into account. A comparison of the profiles of the radiation field measured using a scintillating screen and a CCD camera^48^ with the simulations shows a good agreement, as is evident in Figure S1.

The correction factor for the dose delivered to the cell cultures, with respect to that measured with the ionization chamber, is given by the ratio of the doses obtained from the simulations for both geometries, normalized to the number of particles in each simulation. The combined statistical uncertainty in both doses was less than 0.5%. The systematic uncertainty in the ratio between the two doses is estimated to be approximately 1% because the individual systematic uncertainties cancel each other out to a very large extent. The effects of multiple scattering and of the nuclear interaction in the polystyrene and water are similar and the particle energy at the location of the ionization chamber and at the centre of the cell culture vessel differed by less than 10 MeV, while the variation in the particle energy over the length of the cell culture vessel amounted to about 40 MeV. The variation in this ratio in the cell culture vessel along the direction of the beam is displayed in Fig. 2A. Furthermore, the very good agreement of the results obtained with the two completely independent codes supports our assumption that the systematic error in the dose ratio is small.

An estimate of the dose-averaged linear energy transfer (LET) in the cell culture vessel, which is not easily measured, has been obtained from the GATE simulation. In Fig. 2B, the variation of the dose-averaged LET in the cell culture vessel is shown. The spread between the points is indicative of the statistical accuracy of the simulation.

### Cell lines and cell culture

All cell lines and plasmids have been described previously^27^. Briefly, proteins of interest tagged with Clover—a GFP derivative (Ex λ = 505 nm and Em λ = 515 nm) were transfected into ARPE-19 (human retinal pigmented epithelium, ATCC, CRL-2302). Cells were cultured in DMEM with 4.5 g/L D-glucose, 1 mM sodium pyruvate and 4 mM L-glutamine (Gibco, Life Technologies), supplemented with 100 units/ml of penicillin G (Gibco, Life Technologies), 100 μg/ml of streptomycin (Gibco, Life Technologies) and 10% (v/v) fetal bovine serum (Gibco, Life Technologies). Customized T25 flasks (Thermo Fisher Scientific) were used as cell culture and imaging vessels. As shown in Fig. 4B, the 15 mm-diameter round openings were cut in the centre of the vessel bottoms. The openings were sealed with 25 mm-diameter round glass coverslips (170 μm thickness, no. 1.5H) using a medical-grade silicone kit, and air-dried for 48 h at room temperature. Cells were seeded on the coverslips 24 h before irradiation and incubated at 37 °C and 5% CO_2_.

### Cell imaging and fixation

Real-time imaging was performed using a Nikon C1 inverted confocal fluorescence microscope, mounted in the accelerator beamline (see above for detailed description), equipped with a 40x/0.75 oil immersion objective and a 37 °C incubator (Okolab). The time-lapse series were recorded for 16 min at 2-min intervals. During imaging, the focus was maintained manually, based on phase-contrast image, due to the absence of auto-focusing hardware. Imaging was limited to single, 2D sections of the cell nuclei due to the relatively slow scanning speed of the microscope. After imaging, cells in each vessel were washed twice with PBS (Lonza), fixed using a 3% formaldehyde solution in PBS, and stored in dark at 4 °C. 3D images of the fixed cells expressing RNF168-Clover were acquired in nine areas of coverslips (Fig. 4B, twenty randomly selected cells per area) of two different vessels, with a 63x/1.32 oil-immersion objective mounted on an inverted wide-field Leica DMi8 microscope.

### Image processing and analysis

Individual cells from the 2D real-time confocal imaging series were cropped and stabilized using the ImageJ StackReg plugin. The time of appearance of repair protein foci was then manually determined using the Cell Counter ImageJ plugin. 3D wide-field images of fixed cells were deconvolved using Huygens Professional (SVI Imaging) and maximum-intensity projections were generated using ImageJ. To determine the dose distribution on the coverslips, repair protein foci were counted manually using the Cell Counter ImageJ plugin in twenty, individual, randomly selected cells per area. Data was analyzed and results plotted using GraphPad Prism 8.3.0 (538).

## Supplementary Information

Supplementary Information.
