# Supplementary material for: A simple microscopy setup for visualizing cellular responses to DNA damage at particle accelerator facilities
Source: Sci Rep. 2021 Jul 15;11:14528. doi: 10.1038/s41598-021-92950-1 (PMC8282881; doi:10.1038/s41598-021-92950-1)
Supplement: Supplementary file 1 — Supplementary Information. [file 41598_2021_92950_MOESM1_ESM.pdf]

A simple microscopy setup for visualizing cellular responses to DNA damage at particle accelerator facilities

Haibin Qian[1], Ron A. Hoebe[1], Michel R. Faas[1], Marc Jan van Goethem[2], Emiel R. van der Graaf[2], Christoph Meyer[2], Harry Kiewiet[2], Sytze Brandenburg[2], and Przemek M. Krawczyk[1]*

[1]Department of Medical Biology, Amsterdam University Medical Centers (location AMC) and Cancer Center Amsterdam, The Netherlands
[2]Department of Radiation Oncology, University Medical Center Groningen, University of Groningen, The Netherlands
*to whom the correspondence should be addressed: p.krawczyk@amsterdamumc.nl

Supplementary figures

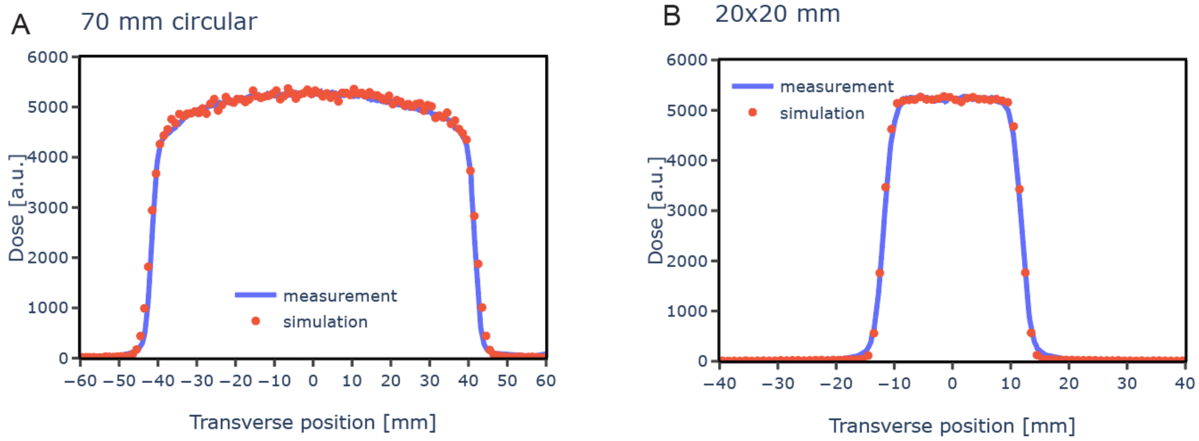

Figure S1. Comparison of the measured (filled line) and simulated (red dots) horizontal profile across the center of the radiation field for (A) the 70 mm circular collimator; (B) the 20 × 20 mm^2 square collimator. The simulations have been normalized to the measurement in the central 10 mm of the radiation field.

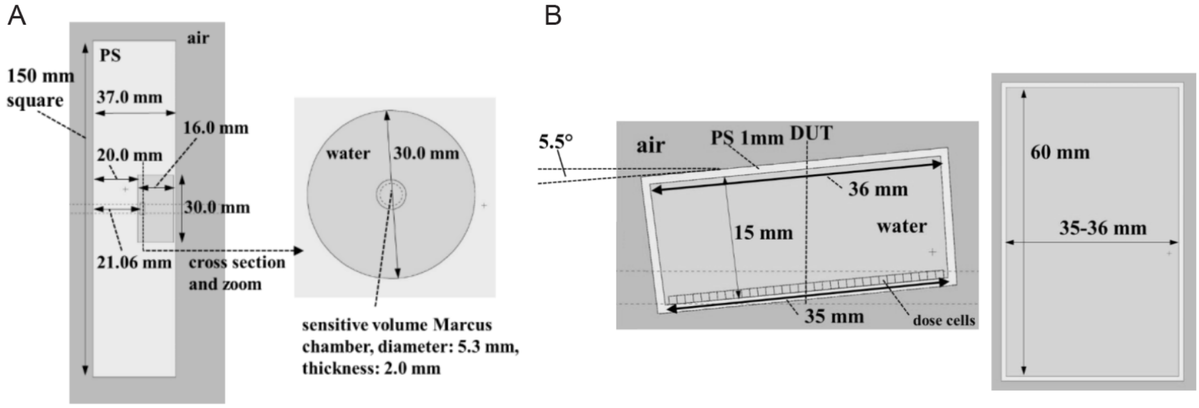

Figure S2. Geometry for the simulation of the dose deposition in the Marcus chamber (A) and in the cell culture vessel (B). Left: side view along the beam direction. Right: top view. The center of the cell culture vessel corresponds to the dashed vertical line marked with DUT.
